# Horizontal augmentation techniques in the mandible: a systematic review

**DOI:** 10.1186/s40729-022-00421-7

**Published:** 2022-05-09

**Authors:** Ralf Smeets, Levi Matthies, Peter Windisch, Martin Gosau, Ronald Jung, Nadine Brodala, Martina Stefanini, Johannes Kleinheinz, Michael Payer, Anders Henningsen, Bilal Al-Nawas, Christian Knipfer

**Affiliations:** 1 Department of Oral and Maxillofacial Surgery, University Medical Center Hamburg-Eppendorf, Hamburg, Germany; 2 Division of “Regenerative Orofacial Medicine”, Department of Oral and Maxillofacial Surgery, University Medical Center Hamburg-Eppendorf, Hamburg, Germany; 3 Mildred Scheel Cancer Career Center HaTriCS4, University Medical Center Hamburg-Eppendorf, Hamburg, Germany; 4 Department of Periodontology, Faculty of Dentistry, Semmelweis University, Budapest, Hungary; 5 Department of Prosthetics and Dental Material Science, Center for Dentistry, Zürich University, Zurich, Switzerland; 6 Periodontics LLC, Chicago, USA; 7 Department of Biomedical and Neuromotor Sciences, Bologna University, Bologna, Italy; 8 Department of Oral and Maxillofacial Surgery, University Medical Center Münster, Münster, Germany; 9 Department of Oral Surgery and Orthodontics, Medical University Graz, Graz, Austria; 10 Department of Oral and Maxillofacial Surgery, Plastic Operations, Mainz University Medical Center, Mainz, Germany

**Keywords:** Augmentation, Horizontal, Lateral, Mandible, Lower jaw

## Abstract

**Purpose:**

Placement of dental implants has evolved to be an advantageous treatment option for rehabilitation of the fully or partially edentulous mandible. In case of extensive horizontal bone resorption, the bone volume needs to be augmented prior to or during implant placement in order to obtain dental rehabilitation and maximize implant survival and success.

**Methods:**

Our aim was to systematically review the available data on lateral augmentation techniques in the horizontally compromised mandible considering all grafting protocols using xenogeneic, synthetic, or allogeneic material. A computerized and manual literature search was performed for clinical studies (published January 1995 to March 2021).

**Results:**

Eight studies ultimately met the inclusion criteria comprising a total of 276 procedures of xenogeneic, allogeneic, or autogenous bone graft applications in horizontal ridge defects. Particulate materials as well as bone blocks were used as grafts with a mean follow-up of 26.0 months across all included studies. Outcome measures, approaches and materials varied from study to study. A gain of horizontal bone width of the mandible with a mean of 4.8 mm was observed in seven of eight studies. All but one study, reported low bone graft failure rates of 4.4% in average.

**Conclusions:**

Only limited data are available on the impact of different horizontal augmentation strategies in the mandible. The results show outcomes for xenogeneic as well as autologous bone materials for horizontal ridge augmentation of the lower jaw. The use of allogeneic bone-block grafts in combination with resorbable barrier membranes must be re-evaluated. Randomized controlled clinical trials are largely missing.

**Supplementary Information:**

The online version contains supplementary material available at 10.1186/s40729-022-00421-7.

## Background

Dental implantology has evolved as an advantageous treatment method for dental rehabilitation and prosthetic restoration of partial or fully edentulous jaws [1, 2]. Sufficient amount and quality of bone remains a determining requirement for successful long-time implant survival and success and to prevent peri-implant disease [3–5]. Physiologic bone loss has been reported in various studies as a logical consequence after tooth extraction [6, 7]. In cases of severe bone loss—mostly due to long time interval since tooth removal, unfavorable load, infection, trauma, or other reasons—bone frequently needs to be augmented prior to or during implant placement [8, 9]. Different augmentation techniques have been developed depending on localization, extent, and configuration of the bone defect [10, 11].

If alveolar bone loss is limited, bone splitting and spreading are useful techniques for adaptation to the local conditions, although this may be difficult to apply in strong cortical bone such as the mandible [12]. Narrow diameter implants have also been proven to serve as possible solutions in horizontally compromised bone [13]. However, in cases of severe horizontal bone loss, horizontal augmentation of the mandibular alveolar ridge may be necessary and can be achieved by a variety of surgical approaches [9]. These procedures include insertion of bone grafts or stimulating bone formation in terms of guided bone regeneration [14–17]. Autologous bone is still regarded as gold standard given its biological properties [9, 18]. However, due to aspects like donor site morbidity, prolonged time of surgery and unpredictable resorption dynamics, a variety of allografts, xenografts and synthetic materials have been introduced into the market and numerous compositions are commercially available. Moreover, these grafts are commonly combined with autologous bone to improve compatibility and combine the advantages of these materials [19]. Additionally, this diversity is extended by different surgical techniques and protocols. Due to the enormous number of materials and techniques, a randomized controlled trial to evaluate and compare all the different approaches would be impossible to conduct. However, there exist scientific reports on the topic that can provide a considerable body of knowledge. For instance, Troeltzsch et al. provide a comprehensive analysis of the available literature for the augmentation of the alveolar ridge [20]. Bone formation in the augmented areas varied from 33.2 ± 14.9% for allogeneic grafts to 56.0 ± 25.6% for mixtures of autogenous and other grafting materials. The authors derive a horizontal gain of 3.7 mm for particulate, compared to 4.5 mm for block grafts. Despite a detailed evaluation of horizontal and vertical dimension, analysis of the donor site (autogenous iliac crest, calvarium, mandible, allogeneic) or the subclassification of the results with regard to the use of membranes and meshes, distinct results for the horizontally compromised lower jaw as recipient site remain elusive.

Furthermore, implant survival and success in concomitantly vs. subsequently placed dental implants are not reported uniformly, so that this aspect remains unclear to date. Thus, the aim of this report was to systematically review the available data and potentially draw conclusions about the efficacy in gaining bone width, implant survival and success rates after or accompanying horizontal ridge augmentation procedures using autologous, xenogeneic, synthetic, or allogeneic materials or combinations of these in cases of bone loss in the lower jaw, which require lateral augmentation. The idea was to systematize approaches and give recommendations on this complex and relevant subject by assessing the efficacy of grafting materials with respect to clinically relevant parameters.

## Results

### Study characteristics

**Fig. 1:**
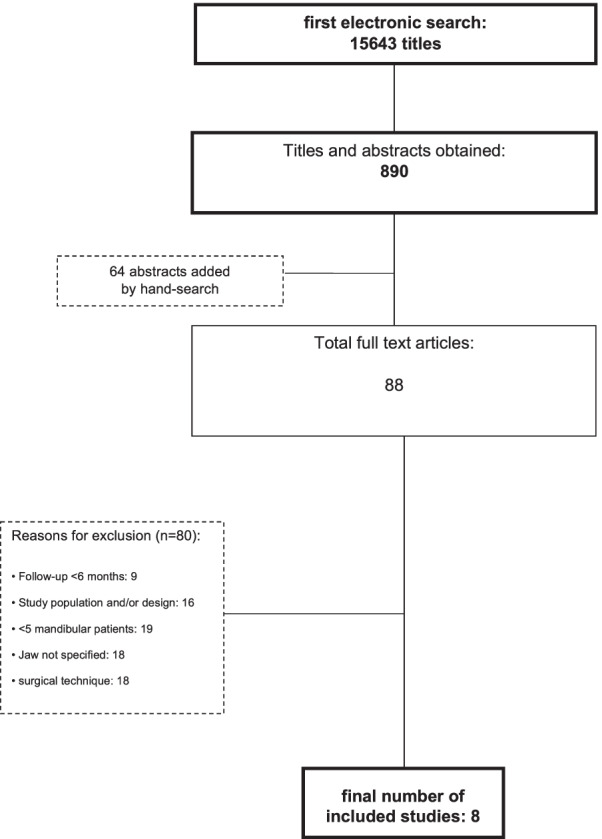
Tree diagram of exclusion/inclusion of studies for this systematic review

The initial electronic search identified a total of 15,643 titles (Fig. 1). 890 studies were selected for further assessment of the abstract after screening of the titles. 64 articles were added by manual search. Out of these, a total of 866 were excluded. Subsequently, 88 full-text articles were obtained, and “Materials and methods” and “Results” sections were investigated. Only eight studies ultimately met the inclusion criteria (Fig. 1). The reasons for exclusion are depicted in Table 1.

**Table 1:** Specification of excluded studies after full-text analysis

Excluded studies	
Surgical technique (e.g., vertical augmentation, split crest):	[14, 21–36]
Maxilla or jaw not specified:	[15–17, 37–52]
< 5 mandibular patients:	[53–71]
Follow-up < 6 months:	[18, 72–81]
Study population or design:	[82–98]

Data synthesis and/or meta-analysis were not performed due to the heterogeneity of the study designs and parameters. Risk of bias of each study is given in Table 2. Information on additional risk of bias across studies is elaborated in “Discussion” section.

**Table 2:** Assessment of risk of bias according to The Cochrane Collaboration’s tool for RCTs, the ROBINS-I tool was applied for prospective cohorts, and the Checklist for Case Series from the Joanna Briggs Institute

	Random sequence generation	Allocation concealment	Blinding of participants and personnel	Blinding of outcome assessment	Incomplete outcome data	Selective reporting	Other bias
Amorfini et al.	+	+	+	+	+	+	+
Nissan et al.	?	?	?	?	+	+	+
Barbu et al.	?	?	?	+	+	+	+
Beitlitum et al.	−	−	−	−	+	+	+
Di Stefano et. al.	−	−	−	−	+	+	+
Schwartz-Arad et al.	−	−	−	−	+	+	+
Silva et al.	−	−	−	−	+	+	+
Urban et al.	−	−	−	−	+	+	+

+: low, −: high, and ?: unclear risk of bias

### Efficacy of augmentation procedures in horizontally resorbed ridges

**Fig. 2:**
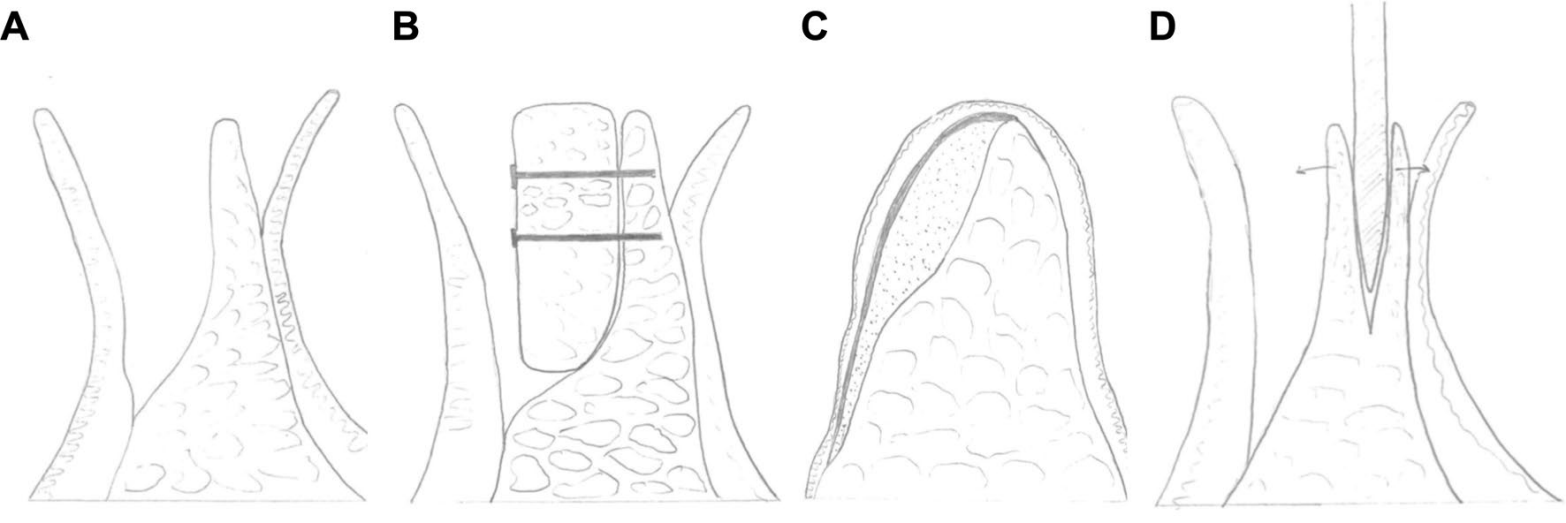
Schematic of horizontal bone loss in the lower jaw after crestal gingival exposure (**A**). Principle of bone-block grafting and fixation with screws (**B**). Depiction of lateral augmentation using particulate bone and membrane placement for coverage (**C**). Gaining of horizontal bone width by surgical splitting of the alveolar ridge (**D**)

**Fig. 3:**
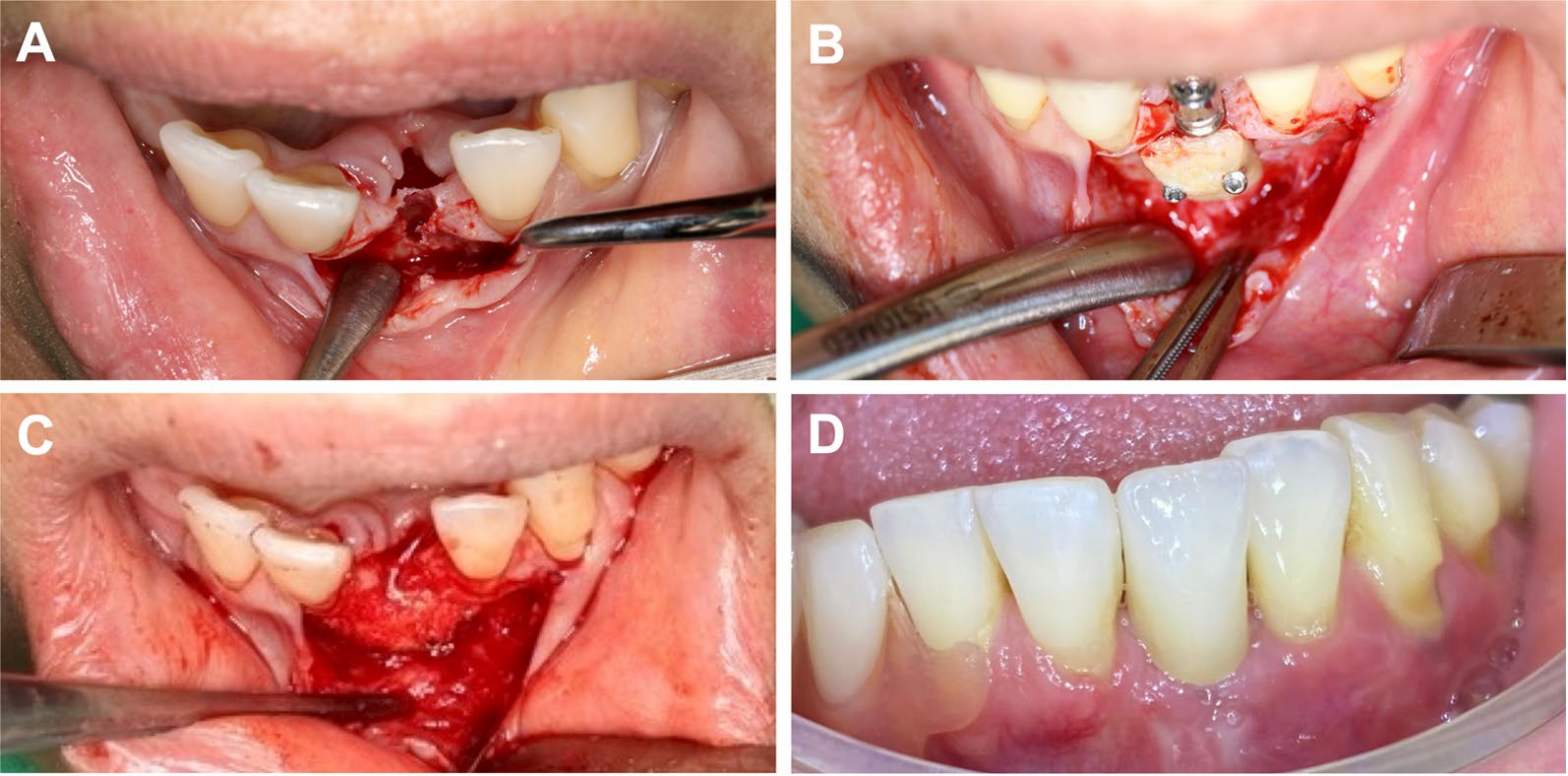
Clinical example of a 41-year-old female patient presenting with missing tooth 31 and consecutive horizontal bone loss (**A**). For dental, functional and esthetic rehabilitation, simultaneous implant placement and lateral augmentation was performed. Implantation of Conelog Progressive Line, harvesting of a retromolar bone block from the mandibular angle with a trephine drill, augmentation and microscrew fixation (1.0-mm steel screws) after rounding of the edges with a burr (**B**). Relining and fitting with particulate bone placement (**C**). After 5 months of healing time, implant exposure and fixation screw removal, sequential abutment fitting and placement of dental crown were performed (**D**)

Eight studies met the inclusion criteria containing a total of 276 procedures of xenogeneic, allogeneic, or autogenous bone graft applications in horizontal ridge defects. Outcome measures, approaches and materials varied from study to study. The mean follow-up was 26.0 months across all included studies, with a maximum mean observation period of 40.5 months, and minimum mean observation period of 6 months. Particulate material as well as bone blocks were used as grafts in the studies (see Figs. 2 and 3).

The autologous block grafts used in the included studies were taken from the mandibular ramus [99, 100] or as bone chips from the posterior mandible/retromolar region [100–102]. A combination of autografts with xenografts or allografts was used in four studies. All augmentation sites were covered, either by membranes or platelet-poor plasma. Table 3 shows the variety of outcome measures used in the included studies.

**Table 3:** Methodological characteristics of the selected studies, the regenerative objective (simultaneous or staged), the types of interventions and measured outcomes

Author	Year	Study design	Setting	Type of augmentation	Regenerative objective	Interventions		Test/control, *n*		Preparation	Outcomes measures
						Test	Control	Patients	Sites		
Amorfini et al. [101]	2014	RCT, parallel, allocation ratio of 1:1, split-mouth model	Private practice	Lateral	Accompanying implant placement	Corticocancellous allograft (iliac crest) + collagen membrane (Biogide®, Geistlich AG) + rhPDGF-BB (GEM 21S®, Osteohealth) or saline solution	Autologous bone chips + bovine bone xenograft (BioOss®, Geistlich) + collagen membrane (Bio-Gide®, Geistlich) + rhPDGF-BB (GEM 21S® Osteohealth) or saline solution	8/8	8/8	Block graft (treatment) and particulate material (control)	BV, RA, BLCT, BOP, PPD, PI, mBL
Barbu et al. [100]	2016	RCT	University clinic	Lateral	Two-staged	Autogenous ramus block (retromolar) + Autogenous particulate bone + PRF membrane (Group 1)	Autogenous ramus block (retromolar) + Autogenous particulate bone chips mixed with xenograft particulate bone + Pericardium membrane (Group 2)	12/12	12/12	Crestal incision, block graft, cortical perforation of recipient bed	RA, IS, BG, EX, PGF, TGF
Beitlitum et al. [117]	2018	Retrospective study	Two periodontal practices, one university dental clinic	Lateral	Two-staged	Particulate mineralized bone allograft (MinerOss, BioHorizons or Maxgraft, Botiss) covered with a cross-linked resorbable collagen barrier membrane	–	15/–	16/–	Crestal incision, particulate allograft	RA, EX, IS, ME, PGF, TGF
Di Stefano et al. [104]	2009	Case series	Private practice	Lateral	Two-staged	Onlay graft of equine spongy bone layer (Osteoplant Flex, Bioteck, Italy) + titanium-reinforced membrane	–	5/5	5/5	Onlay xenograft	HA, PGF, TGF, RA
Nissan et al. [103]	2011	RCT	–	Lateral and vertical	Two-staged	Cancellous allograft + mineralized freeze-dried bone allograft (OraGraft, Lifenet), or bovine bone xenograft (BioOss®, Geistlich) + Membranes: Ossix Plus, OraPharma; Ossix, Biomet; Bio-Gide, Geistlich;	Pre-augmentation measurements of crest width and height	21/–	29/–	Crestal incision, block graft	WG, WR, ME, HR
Schwartz-Arad et al. [99]	2016	Retrospective study	Outpatient surgical center	Lateral and vertical	Two-staged	Autologous intraoral graft + bovine bone xenograft (BioOss®, Geistlich AG) + platelet-rich plasma (PRP) or platelet-poor plasma (PPP)	–	n/a	115/–	Crestal incision, block graft	RA, EX, EXH, EXW, Inf, IS, ISC, ME, PGF, TGF, SP
Silva et al. [105]	2017	Prospective study	University clinic	Lateral and vertical	Two-staged	Fresh-frozen bone-block allograft (femoral epiphyses) from tissue bank (UNIOSS, Brazil) + BBM granules (BioOss, Geistlich, Switzerland) + resorbable collagen membrane (Bio-Gide, Geistlich, Switzerland)	–	20/–	50/–	block allograft, particulate material	RA, EX, PGF, TGF, BV, HA, IS, mBL, SP, WG
Urban et al. [102]	2013	Case series	University Clinic	Lateral	Two-staged	xenograft (inorganic bovine bone-derived mineral, Bio-Oss) + autologous bone graft + resorbable collagen membrane	–	13/–	16/–	particulate material	BV, IS, ISC, PGF, SP, WG, WR

*BOP* bleeding on probing, *BV* bone volume changes, *CAL* peri-implant clinical attachment level, *CCT* controlled clinical trial, *ePTFE* expanded polytetrafluoroethylene, *EX* exposure, *EXH* exposed site height, *EXW* exposed site width, *HA* histological assessment, *IS* implant survival, *mBL* mean marginal bone loss, *ME* membrane exposure, *PGF* partial graft failure, *PI* peri-implant plaque index, *PPD* peri-implant probing depth, *PRF* platelet-rich fibrin, *RA* radiographic assessment, *POP* penetration of the probe, *RCT* randomized controlled trial, *rh-BMP2* recombinant human bone morphogenic protein 2, *rhPDGF-BB* recombinant human platelet-derived growth factor-BB, *SP* success rate procedure, *TGF* total graft failure, *WG* width gain, *WR* width reduction

Even though this review aims to report the results in a standardized way, the wide range of different settings, study protocols and outcome parameters limit conclusions in an evidence-based manner. Hence, only circumscribed quantitative data could be analyzed and this review should be perceived with a narrative approach. The study design ranged from (randomized) controlled clinical trials to pro- and retrospective studies. Particularly, the outcome measures varied extensively in the presented reports. All studies referred to clinical assessment and radiographic imaging when reporting outcome parameters (Table 4).

**Table 4:** Assessment parameters and study results

Author	Assessment method	Initial horizontal width in mm	Final horizontal width in mm	Horizontal gain in mm	Loss in mm	Bone formation in %	bone graft failure in %	Implant survival in % at last follow-up
Amorfini et al. [101]	Clinical assessment; CBCT scan	–	–	5.7	0.2	–	0	100
Barbu et al. [100]	Clinical assessment, CBCT scan	3.5	8.7	5.2			4.2	100
Beitlitum et al. [117]	Clinical assessment, CBCT scan	5.8 ± 0.6	10.0 ± 1.4	4.2 ± 0.9		–	0	100/24 mo
Di Stefano et al. [104]	Clinical assessment, CT scan, OPG, histology, immunohistochemistry	3.9 ± 0.1	7.1 ± 0.2	3.2		35	0	100
Nissan et al. [103]	Clinical assessment; CBCT scan; OPG	–	–	5.6 ± 1.0	0.2 ± 0.2	–	20.7	95.3/37 mo
Schwartz-Arad et al. [99]	Clinical assessment; OPG, CT scan	–	–	–	–	–	3.6	98.5/12 mo 92.5/36 mo 77.5/48 mo
Silva et al. [105]	Clinical, histology, microtomographic morphometry	–	–	4.6 ± 1.3	0.6	31.8	0	96/31.8 mo
Urban et al. [102]	Clinical assessment; periapical radiographs histomorphometry in 9 sites	1.9	7.2	5.3	1.1	31	6.3	100

Complications %: number of patients complication rate in the augmented sites occurring during the observation period; implant survival %: survival rate of implants in the augmented area in percent; horizontal gain (mm): horizontal augmentation result at the end of the observation period in millimeters; horizontal width (mm): horizontal metrics at the end of the observation period in millimeters; loss (mm)/(%): difference between the initially augmented distance and the final result in millimeters/percent; bone formation (%): amount of newly formed bone in the defect area in percent

The majority of the included patients were female. Where specified, individuals were 70 women (72.2%) compared to 27 men (27.8%). The inclusion criteria for augmentation procedures and subsequent implant therapy did also vary, as for instance in some studies smokers were explicitly excluded from the study and in other studies smoking status of the patients was only recorded (Table 5). In all studies, the systemic health status was addressed in the inclusion criteria. However, the parameters ranged from patients being “in good health” to listing of specific systemic medical conditions such as “connective tissue diseases” and “immunodeficiencies”.

**Table 5:** Patient characteristics and implant specification

Author	Gender	Age (y)	Inclusion criteria	Exclusion criteria	Smoking	Systemic condition	Implant placement after augmentation (mths)	Types of implants	Mean follow-up (mths)
Amorfini et al. [101]	–	Median 59.5 (32–72)	Adult patients; bilateral atrophic edentulous areas in the posterior mandible	Systemic diseases affecting the bone metabolism	n/a	No systemic condition affecting bone metabolism	Simultaneous	SLActive; Straumann (Basel, Switzerland)	12
Barbu et al. [100]	13 female; 11 male	Mean 47.8 (24–71)	At least 10-mm residual height, but less than 4.3-mm bucco-lingual dimension	Smoking; uncontrolled systemic disease; active periodontal disease	Excluded	No uncontrolled systemic disease	4	TSV Zimmer Dental Inc (Carlsbad, California, USA)	19.8
Beitlitum et al. [117]	12 female; 3 male	Mean 53 (36–68)	Mandibular partial edentulism, horizontal bone deficiency that prevented implant placement	Presence of uncontrolled periodontal disease involving the residual dentition, an active endodontic condition involving the adjacent teeth	> 10cig/d excluded	No pregnancy or lactation, bone disease or medication interfering with bone metabolism, history of head and neck radiotherapy, metabolic disorders (eg, uncontrolled diabetes mellitus), immunodeficiency	5–7	–	6
Di Stefano et al. [104]	3 female; 2 male	Mean 45.5 (32–59)	Crestal width ≤ 4 mm, crestal height ≥ 10 mm, age > 30 years, controlled oral hygiene and absence of lesions	High degree of bruxism; excessive consumption of alcohol; localized radiation therapy of the oral cavity; inflammatory and autoimmune diseases of the oral cavity; and poor oral hygiene	> 10cig/d excluded	No antineoplastic chemotherapy; blood, liver, and kidney diseases; immunosuppression; corticosteroids and bisphosphonates therapy; pregnancy;	6	XiVE, Dentsply-Friadent, (Mannheim, Germany)	40.5
Nissan et al. [103]	18 female; 3 male	Mean 55.7	Patients with a mandibular alveolar ridge requiring a vertical and lateral augmentation of > 3 mm patients in good health	Contraindications to implant therapy	n/a	Healthy	6	Seven; MIS® (Savion, Israel) or Osseotite; Zimmer Biomet 3i (Warsaw, USA)	37 ± 17
Schwartz-Arad et al. [99]	–	50.3	Edentulous mandibular area with degree of atrophy preventing placement of implants of at least 6 mm in height without the risk of damaging anatomical structures	Poor oral hygiene noncompliance	18.2%	Exclusion of kidney/liver/connective tissue disease, immunodeficiency, chemotherapy, radiotherapy	5	Screw-Vent and Spline; Zimmer Biomet 3i, (Warsaw, USA) or NobelActive and Replace Select, Nobel Biocare (Goteborg, Sweden) or Implant Direct (Zurich, Switzerland)	39.9 ± 30.9
Silva et al. [105]	15 female; 5 male	Mean 51.8 (37–64)	Alveolar ridge width and/or height ≤ 6 mm; > 18 yrs. Good oral health, no active periodontal disease, or occlusal problems	Compromised oral or general health, pregnancy, alcohol abuse, radiotherapy, bisphosphonates	Excluded	Good general and mental health	6	TitamaxCM Cortical, Neodent, (Curitiba, Brazil)	31.8 ± 7
Urban et al. [102]	9 female; 3 male	51.4	Horizontal ridge of 4 mm or less, Cawood Howell Class IV, ability to maintain good oral hygiene	Smokers, patients with alcohol abuse, periodontal disease	Excluded	Systemically and periodontally healthy	6.9	Implants with an anodized TiUnite surface (Brånemark System, Nobel Biocare)	20.9 ± 9.5

The horizontal mandibular bone serving as indication for augmentation procedures in the studies was also reported in various ways. The reported projected bone increase needed for implant placement was 3 mm of bone width [103]. Other groups performed lateral augmentation in case of residual width from < 4 mm [102, 104] to < 6 mm [105]. The article of Schwarz-Arad et al. proposed a need for augmentation if an implant of 6 mm in height could not be placed without the risk of damaging anatomical structures. The horizontal gain of bone width after the healing process was evaluated radiographically in seven studies. A gain of horizontal bone width was reported in seven of eight studies, with a mean of 4.8 mm, ranging from 3.2 to 5.7 mm (Table 4).

No significant difference was found regarding the outcome variables due to the heterogeneity of reporting the data. The use of rhPDGF-BB in one study significantly limited the resorption of augmented bone [101]. This observation was true for both the group having been augmented with corticocancellous allograft of the iliac bone and the control group of bone chips from the retromolar region. Marginal bone loss during the healing period was evaluated in four studies, with a mean of 0.5 mm, and values between 0.2 and 1.1 mm. Histologic findings were reported in three studies [102, 104, 105]. In the study conducted by Urban et al., a xenograft was combined with autologous bone and the mandible was augmented using particulated material. Histological results showed autologous bone in 31.0%, ABBM, remaining xenograft in 25.8% and marrow space in 43.2% of the derived samples. Di Stefano et al. and Silva et al. quantified newly formed bone at 35.0% and 31.8%, respectively. None of the studies reported on implant success rates. Implant survival rates were shown to be above 92.5% in the included studies at 36 months of last follow-up (Table 4). Four studies reported on crestal incision techniques with an implant survival rate ranging from 92.5 to 100.0% at 18.9 to 37 months of follow-up. One study group used a lateral incision for the augmentation and the surgical approach was not specified in the remainder of the reports. The declared implant survival rate was 96% to 100% with a mean follow-up of 10 to 40.5 months.

One study reported up to 3.6% complications concerning the grafted site at the follow-up [101]. Although data of horizontal and combined horizontal–vertical expansion of the atrophic mandible have not been reported separately in the investigated work by Schwartz-Arad et al., the total complication rate of mandibular augmentation was found to be 6.1%. However, only two of the seven complications occurred in solely horizontal augmented sites. Failure of one graft [6.3%] was reported in the study by Urban et al., when looking at the data of mandibular augmented sites. A comparatively high failure rate of six bone grafts was observed in the study by Nissan et al. [20.7%]. Although distinct failure rates in the horizontally augmented mandible are missing, most of the allograft failures [71.0%] in this study occurred in the posterior mandible. Barbu and colleagues reported graft exposure 2 weeks after the augmentation in one patient. Another week later clinical signs of necrosis led to removal of the graft resulting in a failure rate of 4.2% [100]. In conjunction with bone graft failure rates of 0.0% in four reports, the overall average amounts to 4.4% across all studies.

## Discussion

Successful long-time survival and success of dental implants depend on sufficient amount and quality of bone. In case of severe horizontal bone loss, horizontal ridge augmentation of the mandibular ridge can provide optimum conditions for successful implant placement. However, the jaw recipient site (maxilla or mandible) has been shown to influence graft resorption [106]. Therefore, the aim of the present review was to systematically examine the clinical efficacy of augmentation procedures in horizontally resorbed mandibular ridges in terms of horizontal bone gain, implant success and survival after a follow-up period of at least 6 months. The results of this systematic review indicate a high variability in types of interventions to gain horizontal bone width. However, all techniques were able to create a sufficient horizontal bone gain. Implant survival was very good with results exceeding 92.5% between 12 and 36 months of follow-up.

Even though the present review aims to report the results in a standardized way, the wide range of different settings, study protocols and outcome parameters prevent drawing conclusions in an evidence-based manner. Only eight studies met the inclusion criteria and could be considered for this review, which is one of the main limitations of this study. However, the authors chose not to further modify the inclusion criteria to provide sufficient evidence. Additional inherent limitations are different study designs, different materials used, different assessment methods and prominently different outcome measures throughout. Therefore, comparisons from study to study are limited and comprehensive statistical analysis was not feasible. Augmentation procedures always included the use of membranes. In all studies, combinations of materials (autologous bone combined with xenografts or allografts) were used. The minimum bone width to include patients was either not specified or differed from study to study. Only three of the evaluated studies were randomized [100, 101, 103]. None of the studies reported on implant success rates. This results in the demand of further studies particularly focusing on how implants survive. This also includes the claim on reporting prosthetic data that were proposed at the 4th EAO Consensus Conference [107].

As case reports or case series with less than five patients did not meet the criteria for inclusion in this review, a considerable part of the pre-selected publications (19 in total) had to be dismissed in the process of data extraction. An observation period falling below 6 months after augmentation was reported in eight studies and considered too short to evaluate augmentation procedure and implant success. A major shortcoming during thorough data extraction was whether the horizontal ridge augmentation took place in the upper or lower jaw. As it has been reported that the augmentation procedure in the mandible may be correlated with higher complication rates and thus might be less predictable, the need for reporting the data in this distinctive way remains high [108].

Overall results show that all bone grafts that were used in the included studies have the potential to increase horizontal bone width. Even though no analysis could be carried out due to the lack of homogeneous data and outcome results, the descriptive data suggest a slightly better performance of bone blocks for horizontal augmentation of the lower alveolar ridge than for particulate material. In contrast, Urban and colleagues found comparable results on bone block augmentations using particulate autologous bone grafts in combination with allogeneic material (horizontal bone gain of 5.3 mm in mean).

Troeltzsch et al. conducted a comprehensive pooled analysis with regard to clinical efficacy of grafting materials in alveolar ridge augmentation over a weighted mean follow-up of 27.4 months (range 3–168 months) [20]. In this review, the mean follow-up was 26.0 months across all included studies, with a maximum mean observation period of 40.5 months, and minimum mean observation period of 6 months. After augmentation, the weighted mean horizontal gain for all particulate grafting materials was 3.7 ± 1.2 mm, with variation between 2.2 ± 1.2 mm (synthetic) and 4.5 ± 1.0 mm (mixtures of autogenous bone with allogeneic/xenogeneic grafting material) without statistical significance in the work of Troeltzsch et al. The authors derive a horizontal gain of 4.5 ± 1.2 mm for block grafts. Limited to the inclusion criteria of our review, a gain of horizontal bone width of the mandible in seven of eight studies, with a mean of 4.8 mm, ranging from 3.2 to 5.7 mm was observed. All but one study, reported low bone graft failure rates of 4.4% in average. One group of authors, that used bone-block allografts combined with particulate mineralized freeze-dried bone allograft or particulate bovine bone xenografts, reported a failure rate of 20.7% [103]. The reasons were only discussed marginally, and the authors suggested a relation to the localization of the augmentation as all failures had occurred in the posterior mandible. However, a successful use of allografts was stated in further studies that did not meet the inclusion criteria of this review. This suggests that other reasons than the use of allografts might be responsible for the high failure rate in the above-mentioned study [105, 109].

The augmentation procedure and subsequent complication rates may also be associated with the surgical approach. Horizontal ridge augmentation techniques in the mandible have been shown to be very efficient and safe. However, the method of placing narrow-diameter implants in horizontally resorbed alveolar ridges provides an alternative approach when bone loss is limited. Studies showed that results of narrow-diameter implants placed to support single crowns in the posterior region of the jaw did not differ from results of regular implants regarding the outcome parameters of marginal bone level, implant survival and success rates [110]. Moreover, recent studies suggest that narrow-diameter implants (2.75 to 3.25 mm) can successfully be used as minimally invasive alternative to horizontal bone augmentation in the posterior mandible with implant survival rates exceeding 97% [111]. A recent clinical trial over 2 years showed that patients receiving mini implants with shorter diameters of 1.8–2.4 mm had clinical outcomes similar to those of patients receiving conventional dental implants to support overdenture prostheses [112]. These results must be taken into account when considering horizontal ridge augmentation procedures in the mandible. To appropriately evaluate lateral augmentation techniques in the mandible, other approaches like the split bone block technique had to be excluded in the present review. Given the lack of information provided in the studies, no recommendation can be given concerning the surgical approach.

All studies included the use of a barrier membrane. Due to missing control groups without membranes, a beneficial effect of this procedure on horizontal ridge preservation techniques accompanying augmentation is conceivable but cannot be evaluated. However, literature regarding the reconstruction of peri-implant dehiscence defects suggests a beneficial effect of barrier membranes on the degree of defect filling [20]. Long-term follow-up data based on imaging results are not consistently provided. According to the guidelines of Albrektsson et al., the majority of crestal bone remodeling occurs during the first 2 years following implant-loading, therefore long-term (at least 2 years) follow-up of implants inserted in the augmented sites would be advisable [3]. Furthermore, bleeding and deep peri-implant pockets with crestal bone loss are closely related to the faith of newly formed hard tissues, which are reconstructed via GBR. Thus, implant success rate, which depends on change of crestal bone level and the lack of inflammatory signs, may represent a more relevant evaluation method of GBR efficacy than implant survival rate during long-term follow-ups.

Considering the limited number of included studies and the various approaches that were used, it is not possible to give concise recommendations for horizontal ridge augmentation procedures. Data of prospective randomized and controlled clinical trials regarding different horizontal ridge augmentation techniques including longer follow-up intervals, standardized outcome measures and distinguished results for the upper and lower jaw are missing. As implant success was not evaluated in any of the present studies, further investigations must be conducted including data on how implants and prosthetic reconstructions survive over time.

The role of growth factors in horizontal ridge augmentation must be outlined in a more specific manner. Two of the included studies did in fact work with growth factors—rhPDGF and PRP/PPP, respectively. As the present review is not designed to outline the role of growth factors, no evidence-based conclusion can be drawn from the present data. However, it must be kept in mind that these growth factors present a bias in the present study, which must be considered when interpreting the results.

It is obvious that different surgeons prefer different surgical techniques according to their individual expertise. Therefore, a comparison of distinct GBR procedures in studies with a single surgeon in a split-mouth design hardly represents comparable data. Thus, randomized controlled trials might represent an objective approach with clinically relevant results if the procedures are performed by two different surgeons practicing their favorable techniques after randomization. This would filter the advantages against the individual skillset of the surgeon.

The primary objective can be concluded based on quantitative data, but from the secondary objectives only implant survival rate can be answered. It is of high interest whether the biological behavior of augmented sites is similar to pristine bone or not. Furthermore, bone block alone or bone block supported GBR techniques are compared with GBR techniques based on particulate bone and xenograft (composite graft). Due to the difficulties of these procedures, but also due to different extent and morphology of bone defects, it is very likely that the superiority of one therapy cannot be easily concluded. However, a standardized follow-up protocol with particular timing and assessment methods is needed for getting clear results regarding techniques and long-term implant success.

Data extraction and assessment were performed by two authors to reduce reviewer bias. However, this kind of bias cannot be ruled out completely. As there is a possibility that only studies were published with favorable outcomes or significant findings, a publication bias might exist. Accordingly, studies with a follow-up of less than 6 months could have been so-called “cancelled studies” due to a poor outcome and therefore are not recorded by the mentioned search strategy [80, 81]. As the data extraction is based solely on reported outcomes in the present studies, a reporting bias and incomplete outcome data might have been influenced the data assessment. Predominantly, in five of eight studies multiple biases exist regarding randomization and blinding within each study. Only one study by Amorfini et al. is reported to be a randomized clinical trial on the research topic, whereas the study by Nissan et al. uses randomization only for parameters like deficiency filling and the use of membranes [101, 103]. Barbu and colleagues divided patients between the use of particulate graft composition and membrane and the data were de-identified before research analysis [100]. No further specifics on blinding and randomization can be found. Possible risks of bias according to the Cochrane Collaboration’s tool for randomized controlled trials, as well as the ROBINS-I tool for prospective cohorts, and the Checklist for Case Series from the Joanna Briggs Institute are given in Table 2.

## Materials and methods

### Focused question and PICO

Does the outcome of horizontal mandibular augmentation using xenogeneic, synthetic, or allogeneic material differ from the outcome of autologous bone grafts with regard to gained quantity of bone width, implant survival, success and complication rate in patients that underwent resorption of the horizontal alveolar ridge? The PICO Question is depicted in Table 6.

**Table 6:** PICO Question

**P**opulation	Healthy patients that suffered from resorption of the horizontal alveolar ridge after tooth removal with the lack of possibility to place dental implants without alveolar ridge augmentation prior to or accompanying implant placement
**I**ntervention	Horizontal ridge augmentation using autologous, xenogeneic, synthetic, or allogeneic material or combinations of these
**C**omparison	Horizontal augmentation using only autologous bone grafts
**O**utcome variables	Primary outcome: gain of bone width Secondary outcomes: implant survival, success and complication rate

### Search strategy

A computerized literature search (Medline/PubMed and Cochrane Central Register of Controlled Trials (CENTRAL)) was performed for clinical studies, including articles published from January 1st, 1995, up to March 31st, 2021. Additionally, manual search was carried out in: *International Journal of Oral and Maxillofacial Surgery*, *Journal of Oral and Maxillofacial Surgery*, *Journal of Craniomaxillofacial Surgery*, *Journal of Clinical Periodontology*, *Journal of Periodontology*, *Clinical Oral Implants Research*, *International Journal of Oral Maxillofacial Implants*, *Clinical Implant Dentistry and Related Research*, *Journal of Implantology*, *Journal of Biomedical Materials Research*. Furthermore, full-text articles of reviews published between January 2012 and March 2021 were obtained. An additional manual search was performed on these reviews to identify relevant studies. The language was limited to English. Risk of bias for RCTs was assessed in agreement with the Cochrane Collaboration’s tool [113, 114]. For non-randomized studies, the ROBINS-I tool was applied, and the Checklist for Case Series from the Joanna Briggs Institute [115]. The study followed the PRISMA guidelines (see Additional file 1: Table S1) [116] and was prospectively registered in the international prospective register of systematic reviews (PROSPERO) under registration number CRD42018082149 (www.crd.york.ac.uk/PROSPERO/). No contact was made to acquire additional data from the authors of the study. Any data report and subsequent conclusions are made based solely on published reports.

### Search terms

The following search terms were applied: (“xenogeneic bone substitute” OR “synthetic bone substitute” OR “allogeneic bone graft” OR “xenograft” OR “allograft” OR “synthetic material” OR “lateral space” OR “bone graft” OR “guided bone regeneration” OR “alveolar ridge augmentation” OR “dental augmentation” OR “ridge atrophy” OR “GBR” OR “horizontal augmentation success” OR “horizontal ridge augmentation” OR “bone substitute” OR “alveolar bone graft” OR “alveolar bone loss” OR “alveolar resorption”). The search was limited to “human trial”. Accordingly, the MESH Terms “clinical study”, “clinical trial”, “controlled clinical trial”, “randomized controlled trial” were used.

AND

(“dental implants” OR “lower jaw” OR “alveolar ridge” OR “implant site” OR “mandible”). The search was limited to the MESH Terms “Humans” and “Clinical Trial”.

### Inclusion criteria

Publications were considered when all of the following criteria were applicable:

1. Human, clinical trials with a minimum of five patients reported upon.
2. Horizontal augmentation with bone grafts or substitute materials prior to or accompanying implant placement.
3. Follow-up of at least 6 months in mean.
4. Metric outcome measures following surgical intervention.

### Exclusion criteria

Publications limited to (1–4) or containing (5–13) the following were excluded:

1. In vitro studies
2. Animal (preclinical) studies
3. Cadaver studies
4. Case reports and reports based on interviews and charts
5. Vascularized free bone grafts
6. Only soft tissue augmentation
7. Distraction osteogenesis
8. Socket preservation techniques
9. Augmentation procedures after removal of malignant or benign tumors
10. Patients receiving radio- or chemotherapy
11. Results of patients that had been presented in prior studies of the authors
12. Only vertical augmentation procedures
13. Other bone preservation procedures, e.g., splitting or spreading.

### Selection of studies

Two researchers (CK and LM) independently screened the publications derived from the online search based on the inclusion criteria. Subsequently, the abstracts of the selected titles were obtained and screened for meeting the inclusion criteria. If an abstract was not available in the database, the abstract of the printed article was used. Full-text articles of the selected abstracts were obtained. Again, a selection was made based on the inclusion criteria for the full-text articles. For this purpose, “Materials and methods” and “Results” sections of these studies were investigated. No additional data were obtained other than data included in the articles.

### Data extraction and method of analysis

Two researchers independently analyzed all data using data extraction tables (CK and LM). Information on the following parameters was extracted: author(s), year of publication, study setting and study design, patient cohort (age range, mean age, gender, drop outs), follow-up (mean time and range), type of grafting material (autologous, allogeneic, xenogeneic, synthetic), type of fixation, type of graft preparation (particulate vs. block graft), type of barrier material used (collagen based, PTFE, polymer, titanium, none), the regenerative objective (two-staged vs. simultaneous), test/control groups, information on smoking or systemic conditions of the patients as well as the timing of implant placement, the assessment method and complications during or after surgery.

### Outcome parameters

Metric data and clinical outcome on the following parameters were extracted, as indicated:

- horizontal width (mm)
- horizontal gain (mm)
- loss of augmented bone width (%)
- complication rate of surgical and post-surgical complications (%)
- bone formation (%)
- implant survival rate (%).

The data extraction was defined as follows:

- Complications (%): complication rate in the augmented sites occurring during the observation period in percent.
- Implant survival (%): survival rate of implants in the augmented area in percent.
- Horizontal width I (mm): horizontal bone width before augmentation in mm.
- Horizontal width II (mm): entire horizontal bone width at the end of the observation period in mm.
- Horizontal gain (mm): horizontal result of gained bone in mm at the end of the observation period.
- Loss (mm)/(%): difference between the initially augmented bone width and the entire horizontal bone width in millimeters/percent.
- Bone formation (%): the bone formation rate is the mineral apposition rate multiplied with the surface area undergoing bone formation. Amount of newly formed bone in the defect area was calculated in percent.

## Conclusions

A variety of approaches and materials exist for the reconstruction of bone width of the mandible. Only limited evidence with risk of bias is available on the impact of using particular grafts or bone blocks for horizontal augmentation in the lower jaw. The results of this review show outcomes for xenogeneic as well as autologous bone materials for horizontal ridge augmentation of the lower jaw. The use of allogeneic bone-block grafts in combination with resorbable barrier membranes must be re-evaluated. Data of randomized controlled clinical trials indicating superiority of specific horizontal ridge augmentation procedures in the lower jaw are still missing and worthwhile being investigated.

## Supplementary Information

**Additional file 1: Table S1.** PRISMA 2020 checklist.

## Data Availability

Not applicable.

## Abbreviations

ABBM: Anorganic bovine bone-derived mineral
BOP: Bleeding on probing
BV: Bone volume changes
CAL: Peri-implant clinical attachment level
CCT: Controlled clinical trial
CENTRAL: Cochrane Central Register of Controlled Trials
ePTFE: Expanded polytetrafluoroethylene
EX: Exposure
EXH: Exposed site height
EXW: Exposed site width
GBR: Guided bone regeneration
HA: Histological assessment
IS: Implant survival
mBL: Mean marginal bone loss
ME: Membrane exposure
PGF: Partial graft failure
PI: Peri-implant plaque index
PICO: Population, Intervention, Comparison, Outcome variable
PPD: Peri-implant probing depth
PRF: Platelet-rich fibrin
RA: Radiographic assessment
POP: Penetration of the probe
PRP: Platelet-rich plasma
RCT: Randomized controlled trial
rh-BMP2: Recombinant human bone morphogenic protein 2
rhPDGF-BB: Recombinant human platelet-derived growth factor-BB
SP: Success rate procedure
TGF: Total graft failure
WG: Width gain
WR: Width reduction
